# Uncompetitive Allosteric Inhibition of PTP1B by BP-1-102 Reveals a Potential Dual-Target Strategy toward the PTP1B–STAT3 Oncogenic Axis: Biochemical and Computational Evidence

**DOI:** 10.1021/acsmedchemlett.6c00242

**Published:** 2026-06-12

**Authors:** Martín González-Andrade, Rodolfo A. Lizárraga-Valadez, Alejandro Sosa-Peinado, Francisco Cortés-Benítez, Nathaly Vasquez-Martínez

**Affiliations:** † Laboratorio de Biosensores y Modelaje Molecular, Departamento de Bioquímica, Facultad de Medicina, 61589 Universidad Nacional Autónoma de México, Ciudad de México 04510, México; ‡ Laboratorio de Fisicoquímica e Ingeniería de Proteínas, Departamento de Bioquímica, Facultad de Medicina, Universidad Nacional Autónoma de México, Ciudad de México 04510, México; § Laboratorio de Síntesis y Aislamiento de Sustancias Bioactivas, Departamento de Sistemas Biológicos, División de Ciencias Biológicas y de la Salud, Universidad Autónoma Metropolitana—Unidad Xochimilco, Ciudad de México 04960, México

**Keywords:** protein tyrosine phosphatase 1B, PTP1B inhibitors, allosteric inhibitor, molecular dynamics, umbrella sampling, dual-target cancer therapy

## Abstract

Selective PTP1B inhibitors paradoxically potentiate oncogenic STAT3 signaling by restoring JAK2 activity, underscoring the need for dual-target strategies. Here we report that BP-1-102, an orally bioavailable STAT3 SH2 domain inhibitor, also potently inhibits PTP1B with IC_50_ values of 5.32 μM (hPTP1B_1–400_) and 11.7 μM (hPTP1B_1–285_), with ≥37.7-fold selectivity over TCPTP. Kinetic analysis identified an uncompetitive mechanism, suggesting inhibitor binding to the enzyme–substrate complex at a site distal to the active site. Docking and 500 ns molecular dynamics simulations revealed a dual-anchor binding mode involving the 113–123 loop (Cys121) and the proline-rich C-terminal disordered region, a structural interface absent in TCPTP. Umbrella sampling confirmed favorable passive membrane permeability through a tumor-mimetic bilayer (Δ*G* = −13.83 kcal/mol). These results propose BP-1-102 as a candidate for dual-function inhibitor of the PTP1B–JAK2–STAT3 oncogenic axis, providing a physicochemical and mechanistic rationale for its further evaluation in tumors where both targets are coactivated.

## Graphical abstract

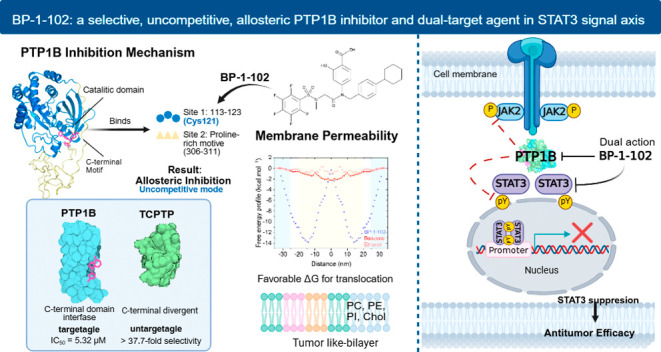

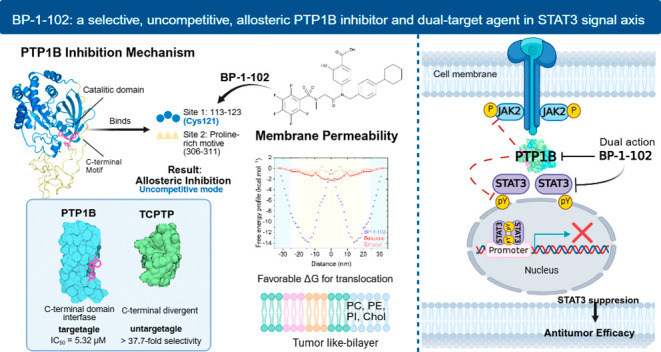

Protein tyrosine phosphatase 1B (PTP1B) is an important negative regulator of insulin and leptin signaling, acting by directly dephosphorylating the insulin receptor (IR), Janus kinase 2 (JAK2), and downstream effectors, including signal transducer and activator of transcription 3 (STAT3).^1–3^ Beyond its established associations with type 2 diabetes and obesity, aberrant PTP1B activity has been linked to tumor progression across various cancer types.^4,5^ PTP1B-mediated modulation of cell growth, survival, and invasion provides a mechanistic bridge between systemic metabolic dysfunction and the hallmarks of cancer, positioning this phosphatase as an emerging target within the metabolic-oncogenic axis.^6,7^ PTP1B-mediated dephosphorylation of the IR at Tyr1158, Tyr1162, and Tyr1163 directly disrupts downstream signaling through insulin receptor substrate proteins IRS-1 and IRS-2.^8^ This prevents the recruitment of phosphoinositide 3-kinase (PI3K) to IRS-1, attenuating Akt activation and its downstream effectors, including mTORC1 and FOXO transcription factors, which regulate glucose uptake, glycogen synthesis, and cellular survival.^8^ In oncogenic contexts, this same axis acquires pathological relevance. While hyperactivation of the PI3K/Akt/mTORC1 pathway is a hallmark of tumor progression,^9^ PTP1B-mediated attenuation of IRS signaling establishes a paradoxical regulatory node, allowing the phosphatase to simultaneously suppress metabolic insulin action and modulate proliferative pathways depending on cellular context.^6^ However, despite decades of effort, no PTP1B inhibitor has achieved clinical approval, largely due to selectivity challenges against closely related phosphatases and poor membrane permeability of compounds targeting the active site.^10^

STAT3 remains a hallmark of oncogenic signaling in human cancers.^11,12^ Following phosphorylation at Tyr705 by JAK kinases, STAT3 dimerizes and translocates to the nucleus to drive the expression of genes governing proliferation, survival, and immune evasion.^13^ This cascade is directly regulated by PTP1B, which dephosphorylates JAK2 and thereby modulates STAT3 activation upstream.^14^ Simultaneous inhibition of both proteins could thus yield a coordinated antitumor response that surpasses the efficacy of single-target strategies, particularly in solid tumors like hepatocellular carcinoma or breast cancer, where both PTP1B and STAT3 are constitutively coactivated.^15,16^ Therefore, interaction between PTP1B and STAT3 signaling presents a significant challenge.

Trodusquemine (MSI-1436), a selective allosteric inhibitor of PTP1B, despite its impressive 200-fold selectivity of TCPTP, was shown to enhance rather than suppress STAT3 phosphorylation in HepG2 cells and hypothalamic tissue,^17^ reflecting the loss of PTP1B-mediated negative regulation over JAK2 phosphorylation. This paradox underscores the clinical need for compounds that inhibit PTP1B without amplifying oncogenic STAT3 signaling.

BP-1-102, an orally bioavailable small molecule originally developed as a STAT3 inhibitor through selective engagement of its SH2 domain (*K*_d_ = 504 nM),^18^ offers a mechanistically distinct opportunity to address this paradox. When we assessed BP-1-102 against human PTP1B, we found that the compound inhibits PTP1B potently and selectively, with IC_50_ values of 5.32 ± 0.9 μM toward the full-length construct (*h*PTP1B_1–400_) and 11.7 ± 0.8 μM in the truncated catalytic domain (*h*PTP1B_1–285_) (Figure 1A,B). The observed 2-fold difference in potency between the constructs suggests that the C-terminal disordered region contributes to inhibitor recognition. This pattern is characteristic of inhibitors that interact with sites distal to the catalytic cysteine, aligning with the allosteric and uncompetitive binding modes previously reported for this regulatory region.^19–22^

**Figure 1:**
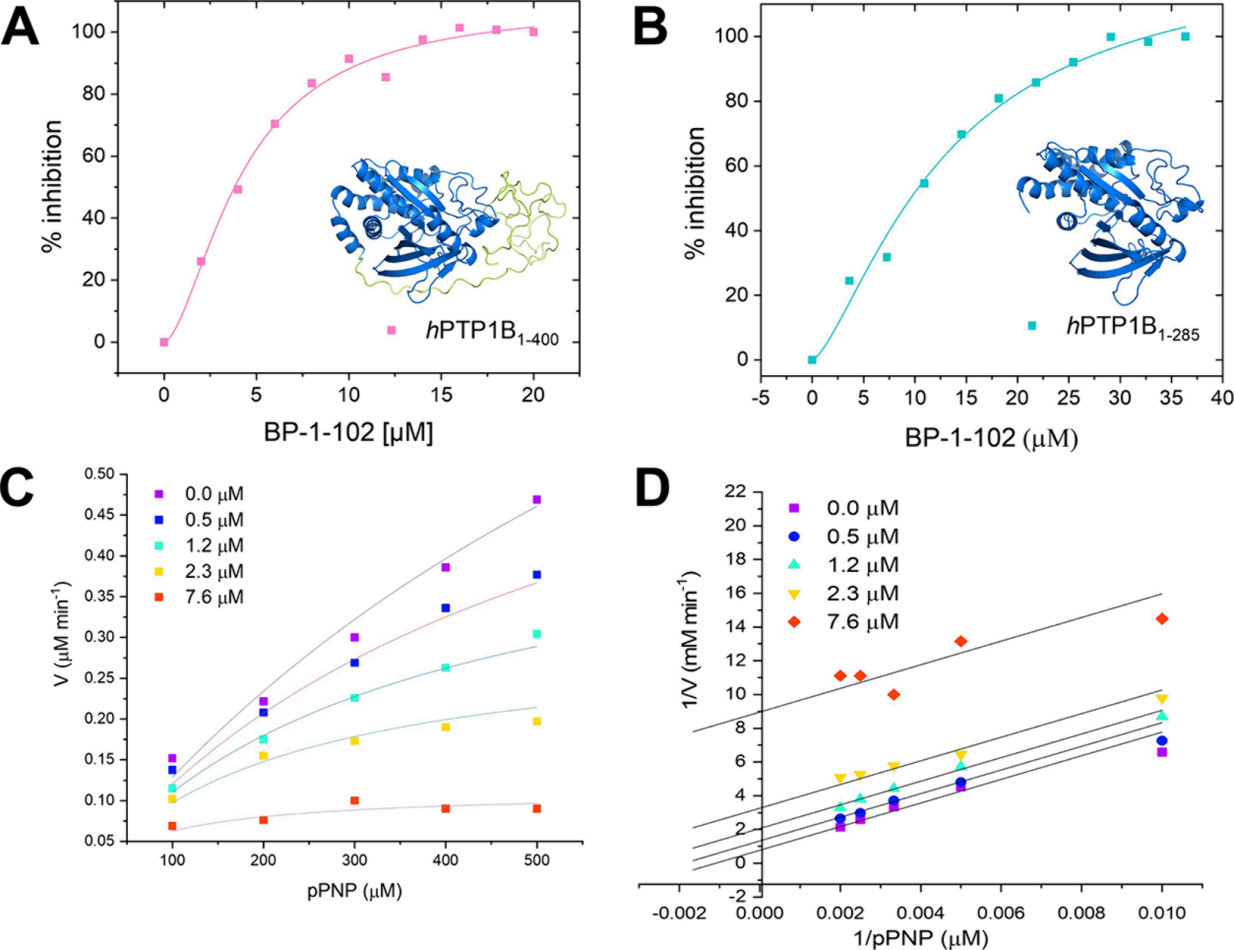
Inhibition of human PTP1B by BP-1-102. Dose–response curve for (A) *h*PTP1B_1–400_ and (B) *h*PTP1B_1–285_. Shown 3D representations of each construct, with the intrinsically disordered C-terminal region highlighted in green. (C) Michaelis–Menten kinetics of *h*PTP1B_1–400_ in the presence of increasing BP-1-102 concentrations (0–7.6 μM). D) Lineweaver–Burk transformation of kinetic data consistent with uncompetitive inhibition (*K*_*ia*_ = 0.72 ± 0.09 μM; *V*_max_ = 1.30 ± 0.16 μM min^–1^; *K*_m_ = 910.99 ± 151.35 μM; *R* ^2^ = 0.99).

Kinetic analysis revealed that BP-1-102 acts as an uncompetitive inhibitor of PTP1B. Nonlinear regression analysis of initial velocity data fitted to competitive, noncompetitive, uncompetitive, and mixed inhibition models identified the uncompetitive model as the best fit (*R* ^2^ = 0.989), yielding a *K*_*ia*_ of 0.72 ± 0.09 μM, a *V*_max_ of 1.30 ± 0.16 μM min^–1^, and an apparent *K*_m_ of 910.99 ± 151.35 μM (Figure 1C,D). Unlike competitive inhibitors, whose potency diminishes at high substrate concentrations, uncompetitive inhibitors increase efficacy as enzyme–substrate occupancy rises. Therefore, BP-1-102 would be expected to act more potently under conditions of high PTP1B activity, a property particularly advantageous in tumor microenvironments, where this phosphatase is overexpressed and constitutively engaged with its substrates.

Given that PTP1B and TCPTP share a 74% identical catalytic domain,^23^ we evaluated the selectivity of BP-1-102 under identical assay conditions. Even at concentrations as high as 200 μM, BP-1-102 did not inhibit TCPTP, and enzyme activity remained at baseline across the concentration range. This resulted in a selectivity index of at least 37.7-fold. The remarkable selectivity of BP-1-102 for PTP1B over TCPTP strongly suggests a mechanism involving regions beyond the active site. This discrimination is crucial because it ensures robust target interaction of the intended enzyme while mitigating adverse effects often associated with cross-reactivity with homologous phosphatases.^24^

To provide a structural rationale for the observed selectivity and uncompetitive inhibition mode, we performed a docking against *h*PTP1B_1–400_. This strategy resulted in a BP-1-102 binding energy of −8.38 kcal/mol. The docking pose showed a network of interactions involving residues from both the catalytic domain and the C-terminal region, including hydrogen-bonding Arg311, hydrophobic contacts (Tyr46, Leu88, Pro89, Leu119, and Pro310), polar interactions (Asn90), and electrostatic contacts (Arg45, Arg47) (Figure 2). Validation of the docking protocol by redocking a cocrystallized allosteric inhibitor yielded a best-pose RMSD of 1.77 Å relative to the crystallographic reference and a predicted affinity of −8.55 kcal/mol, confirming the accuracy of the protocol before application to BP-1-102 (Supporting Information Figure S1).

**Figure 2:**
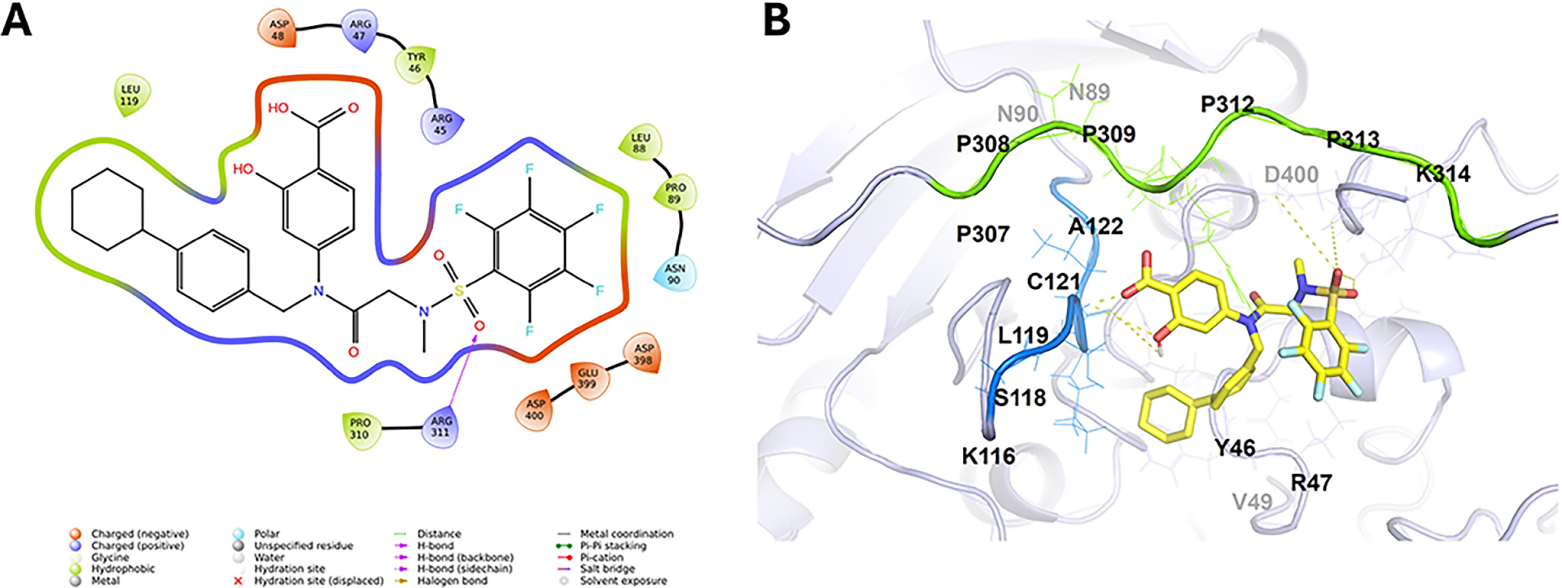
Docking of BP-1-102 to *h*PTP1B _1–400_ at the interface between the catalytic domain and the intrinsically disordered C-terminal region. (A) 2D ligand interaction diagram showing contact with catalytic domain residues (Arg45, Tyr46, Arg47, Asp48, Leu88, Pro89, Asn90) and C-terminal residues (Pro310, Arg311, Asp398, Glu399, Asp400). (B) 3D binding pose highlighting the dual-anchor interaction network. The 113–123 loop (blue) and the proline-rich C-terminal segment (green) flank the ligand (yellow sticks), together with catalytic domain residues Tyr46 and Arg47. Docking was performed using a search box deliberately positioned outside the catalytic site. Binding energy = – 8.38 kcal/mol.

To evaluate the stability and dynamics of the predicted binding mode, 500 ns molecular dynamics simulations (MD) were performed for *h*PTP1B_1–400_ in both its apo and BP-1-102-bound states (holo). Throughout the trajectory, the RMSD of *h*PTP1B_1–400_ in the apo system remained stable, whereas the complex showed a progressive increase, reaching approximately 10 Å after around 230 ns (Figure 3A). Instead of structural instability, the observed divergence reflects a ligand-induced conformational transition. This is corroborated by the convergent radius of gyration (*R*_g_) in both systems (Figure 3B), which shows that BP-1-102 binding does not compromise the overall compactness of the protein. Per-residue RMSF analysis revealed that BP-1-102 increases the conformational flexibility of the full-length protein. Residues 319–334 and 343–376 in disordered C-terminal regions showed the most pronounced ligand-induced flexibility increase (Figures 3C, S2), suggesting potential conformational rearrangements of proline-rich motifs of helices α8 and α9. This heightened flexibility is consistent with the uncompetitive inhibition mechanism; by interacting with the enzyme–substrate complex, the inhibitor modulates the C-terminal regulatory region without locking it into a rigid conformation.

**Figure 3:**
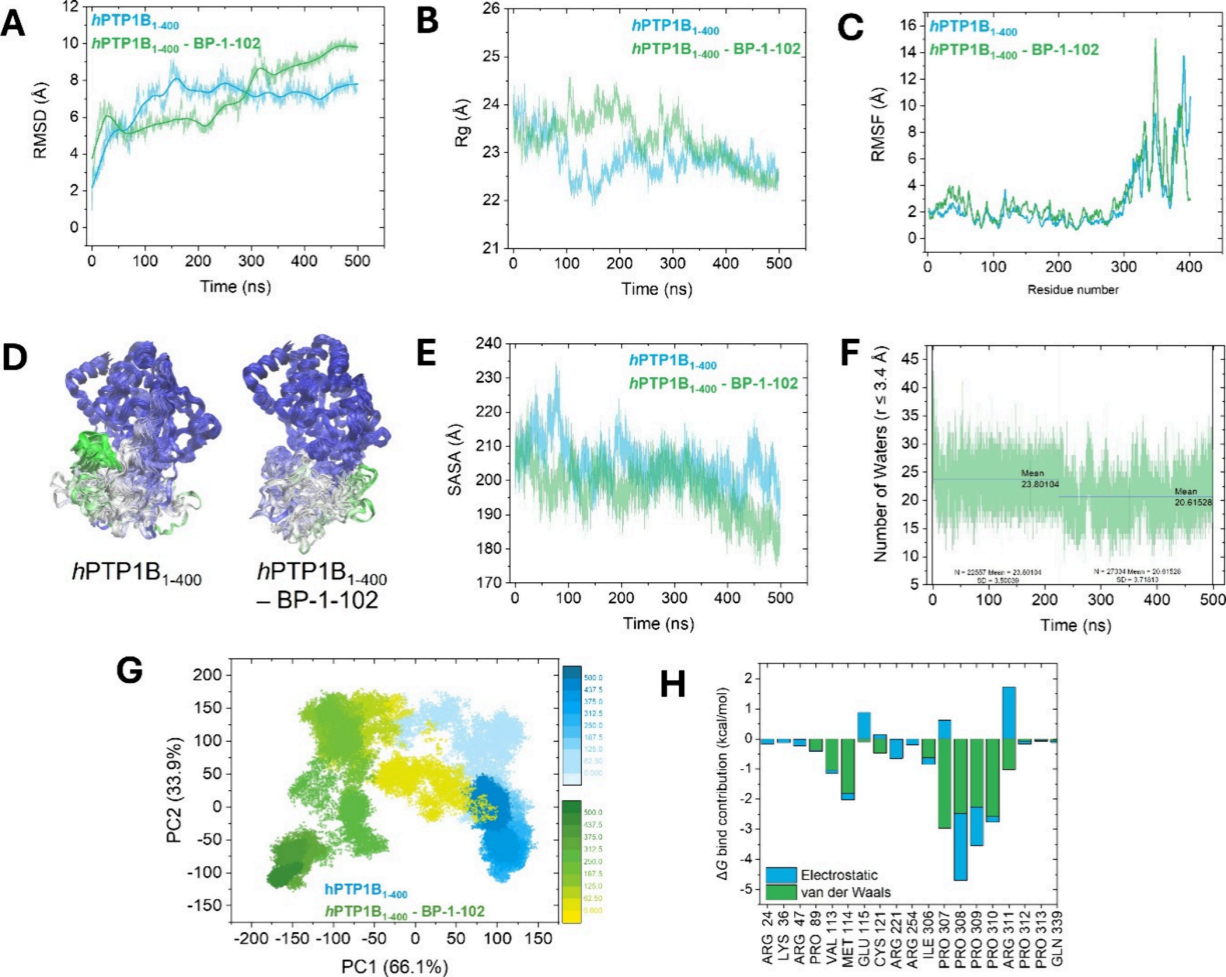
Structural and conformational analysis of the *h*PTP1B_1–400_ by molecular dynamics simulations. Comparative 500 ns MD analysis of *h*PTP1B_1–400_ alone and in complex with BP-1-102. (A) Backbone RMSD, (B) radius of gyration (*R*_g_), (C) per-residue Cα RMSF, (D) MDLovofit conformational flexibility analysis. Blue and green regions represent rigid (∼70% persistence) and flexible (∼30% persistence) regions, respectively. (E) Solvent-accessible surface area (SASA). (F) Number of water molecules within 3.4 Å of BP-1-102. (G) Principal component analysis (PCA) of the combined apo/holo trajectory. Color gradients represent simulation time (0–500 ns). (H) Per-residue binding free energy decomposition (MM-GBSA). Electrostatic and van der Waals contributions are shown for residues.

To assess whether these dynamic changes extend to the catalytic core or remain confined to the C-terminal, analyses were performed on residues 1–300 within the same trajectory. Structural metrics, including RMSD, *R*_g_, and SASA, confirmed that the catalytic domain remained stable and undisturbed by ligand binding (Figure S3). RMSF fluctuations reflected this global stability, except for localized rigidification at residues 117–121 that point to a targeted anchoring of BP-1-102 to this loop. To assess the global collective motions of the full-length protein, MDLovofit analysis was conducted on both *h*PTP1B_1–400_ systems. The C-terminal region exhibited decreased collective mobility in the complex compared to the apo protein (Figure 3D). Therefore, we hypothesize that BP-1-102 acts as a dynamic modulator of the C-terminal region, limiting large-scale collective motions while maintaining localized flexibility within specific segments.

Solvent-accessible surface area (SASA) profiles showed a lower surface exposure for the complex compared to the apo protein across the trajectory (Figure 3E). Consistent with this reduced surface exposure, tracking of the solvent shell revealed that the average number of water molecules within 3.4 Å of BP-1-102 decreased from 23.8 ± 3.1 to 20.8 ± 3.8 at around 230 ns (Figure 3F). This desolvation event potentially reflects a conformational transition linked to the observed RMSD and a gradual tightening of ligand–protein interactions, in which the displacement of interfacial water molecules would provide a favorable entropic contribution to the binding free energy.

Principal component analysis (PCA) confirmed that the apo and holo systems occupy entirely distinct conformational subspaces, demonstrating a ligand-induced shift in the sampled conformational ensemble. Unlike the flexible apo system, which explores a widespread conformational landscape, the complex is confined to a more compact ensemble (Figure 3G). Individual PCA calculations further reveal how this shift reduces the accessible conformational space of *h*PTP1B_1–400_. Specifically, the apo protein exhibits multidirectional fluctuations shared between PC1 (64%) and PC2 (36%), whereas the complex concentrates 78.7% of its total structural variance into PC1 (Figure S4).

Analysis of ligand–protein contacts within 4 Å over the full trajectory confirmed the persistence of the binding (Figure S5). The loop spanning residues 113–123, including Cys121, maintained consistent contact with BP-1-102 throughout the simulation, with additional interactions with the proline-rich segment 306–311 and with the catalytic domain residues Arg45 and Pro89. The involvement of Cys121 is highly relevant, as this residue, although located outside the catalytic site, participates in allosteric communication with His214, adjacent to the active-site nucleophile Cys215, through a network of noncovalent interactions.^25^ Its selective modification has been demonstrated to decrease *V*_max_ without altering *K*_m_ ^26^ a kinetic profile similar to the uncompetitive inhibition reported here. The extended simulation identified additional interactions with C-terminal residues 350–362, a region that includes the α8/9 helices, which exhibited increased RMSF upon ligand binding. Dynamic and contact analyses collectively demonstrate a more extensive binding than initially anticipated from docking alone, encompassing both the catalytic domain–C-terminal interface and distal regions of the disordered C-terminus.

To quantify binding energy, MM/GBSA calculations were performed over the 500 ns trajectory, yielding a Δ*G*_bind_ of −27.30 ± 7.57 kcal/mol. The individual energy contributions to this affinity were identified by residue-by-residue energy decomposition. This analysis showed three primary interaction hubs: Pro89, the catalytic loop region (Val113, Met114, and Cys121), and a continuous C-terminal segment spanning Ile306-Arg311, which are predominantly stabilized by van der Waals forces (Figures 3H and S6). These results are consistent with the binding mode identified by molecular docking and confirm the key residues governing the *h*PTP1B_1–400_-BP-1-102 interaction.

Considering this potential, we investigated whether the compound demonstrated adequate membrane permeability to access the intracellular environment of PTP1B/STAT3-overexpressing cells. To address this question, we calculated the free energy profile of BP-1-102 using a tumor membrane mimetic model (POPC:POPE:POPI:cholesterol, 3:3:0.8:3.2) (Figure 4A). Umbrella sampling analyses revealed a favorable energetic profile for BP-1-102, characterized by a double-minimum profile with Δ*G*_min_ = −13.83 kcal/mol at the lipid headgroup–tail interface (*z* ≈ ±14 Å). This position reflects the preferential stabilization of this amphipathic molecule at the polar/apolar boundary rather than at the hydrophobic core, consistent with the reorientation and anchoring behavior reported for larger amphipathic molecules.^27^ This contrasts with the single-minimum profiles of benzene (Δ*G*_min_ = −2.12 kcal/mol) and ethanol (Δ*G*_min_ = −0.98 kcal/mol), both centered at the bilayer midplane and consistent with passive partitioning of small nonpolar molecules (Figure 4B). Importantly, no significant energy barrier to membrane translocation was observed for BP-1-102 relative to the aqueous phase (ΔΔ*G* ≈ 0 kcal/mol), consistent with passive permeation for amphipathic drug-like molecules.^28^ Together, these results suggest that BP-1-102 can reach intracellular targets, such as PTP1B and STAT3, offering a theoretical rationale for the observed activity in tumor cell environments.

**Figure 4:**
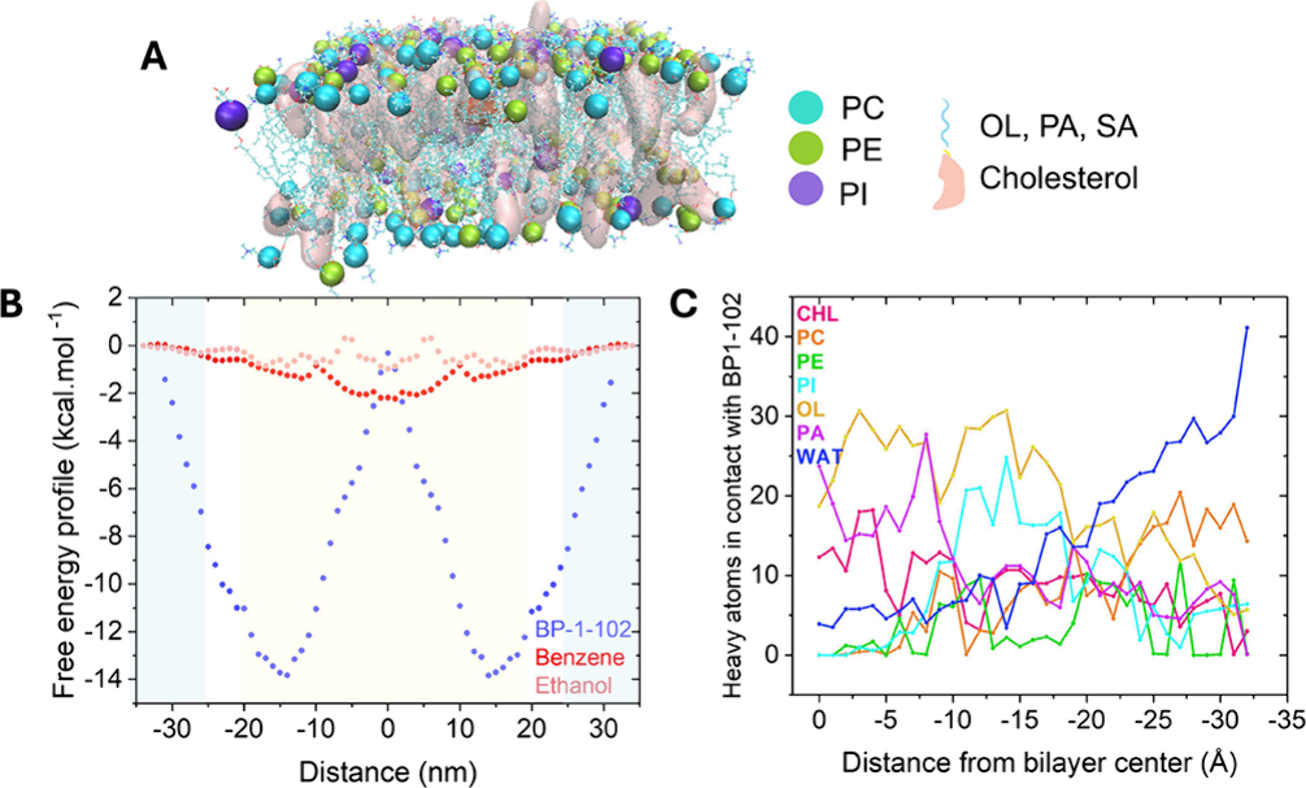
Membrane permeation of BP-1-102 across a tumor membrane mimetic model. (A) Representative view of the simulated lipid bilayer. Membrane was constructed with PACKMOL-Memgen, composed of POPC:POPE:POPI:cholesterol at molar ratios of 3:3:0.8:3.2, respectively. Colored circles represent the phospholipid heads to distinguish each type: phosphatidylcholine (turquoise), phosphatidylethanolamine (lime), phosphoinositol (purple), and cholesterol (pink). The lipid tails include oleoyl (18:1), palmitoyl (16:0) and saturated fatty acid (SA) chains. (B) Free energy profiles for BP-1-102 (blue), benzene (red), and ethanol (light red) across a lipid bilayer tailored to reflect tumor membrane composition, as determined by umbrella sampling. Yellow and blue shaded regions represent the hydrophobic core and the aqueous environment, respectively. (C) Number of heavy atoms of each lipid component in contact with BP-1-102 as a function of distance from the bilayer center, illustrating the preferential interactions established during membrane permeation. CHL, cholesterol; PC, phosphatidylcholine; PE, phosphatidylethanolamine; PI, phosphoinositol; OL, oleoyl; PA, palmitoyl; WAT, water.

The contact profile of BP-1-102 showed a continuous chemical transition from the bilayer center to the aqueous phase (Figure 4C). Deep within the hydrophobic core (*z* < 10 Å), the ligand interacts almost exclusively with the acyl chains, oleoyl and palmitoyl, and cholesterol. As the ligand moves toward the interfacial region (*z* ≈ 10–20 Å), it maintains these hydrophobic contacts while progressively engaging polar head groups (PC, PE, PI) and initial water molecules. Beyond *z* > 20 Å, lipid contacts decrease, and water contacts rise, reflecting the transition to the aqueous phase. This contact profile supports an amphipathic anchoring mechanism where the hydrophobic moieties of BP-1-102 engage the membrane interior while its polar groups remain accessible to the headgroups. This spatial arrangement prevents the molecule from partitioning into the highly ordered hydrophobic core, stabilizing it at the interface rather than penetrating deeper into the cholesterol-ordered hydrophobic core.

Collectively, the kinetic, computational, and membrane permeability data reported here support a mechanistic model in which BP-1-102 acts as an uncompetitive, allosteric inhibitor of PTP1B. This inhibition is mediated through a previously undercharacterized interface involving residues 113–123 and the proline-rich C-terminal region, a binding mode that is structurally distinct from classical α6/α7 allosteric sites. This dual-anchor binding mode restricts the conformational ensemble of *h*PTP1B_1–400_, as evidenced by PCA and MDLovofit analyses, while selectively engaging a structural interface absent in TC-PTP, thereby providing a mechanistic basis for the observed >37-fold selectivity. Furthermore, its favorable passive permeability across a tumor-mimetic membrane explains the cellular accessibility of BP-1-102 in oncogenic environments where PTP1B and STAT3 are coactivated.

In addition to its inhibitory effect on PTP1B, the established antiproliferative activity of BP-1-102 in STAT3-dependent models supports the notion that BP-1-102 disrupts the PTP1B–JAK2–STAT3 signaling axis at two distinct nodes, as proposed in Scheme 1. In this pathway, PTP1B dephosphorylates JAK2 at Tyr1007/Tyr1008, attenuating its kinase activity and downstream STAT3 phosphorylation at Tyr705, the event required for STAT3 dimerization, nuclear translocation, and transcriptional activation of pro-survival genes like Bcl-2, cyclin D1, and c-Myc.^29,30^ However, in malignancies where PTP1B and STAT3 are constitutively coactivated, this axis establishes a self-reinforcing oncogenic loop. In this context, STAT3 upregulates PTP1B expression, which further suppresses JAK2 while paradoxically maintaining basal STAT3 signaling through feedback mechanisms.

**Scheme 1:**
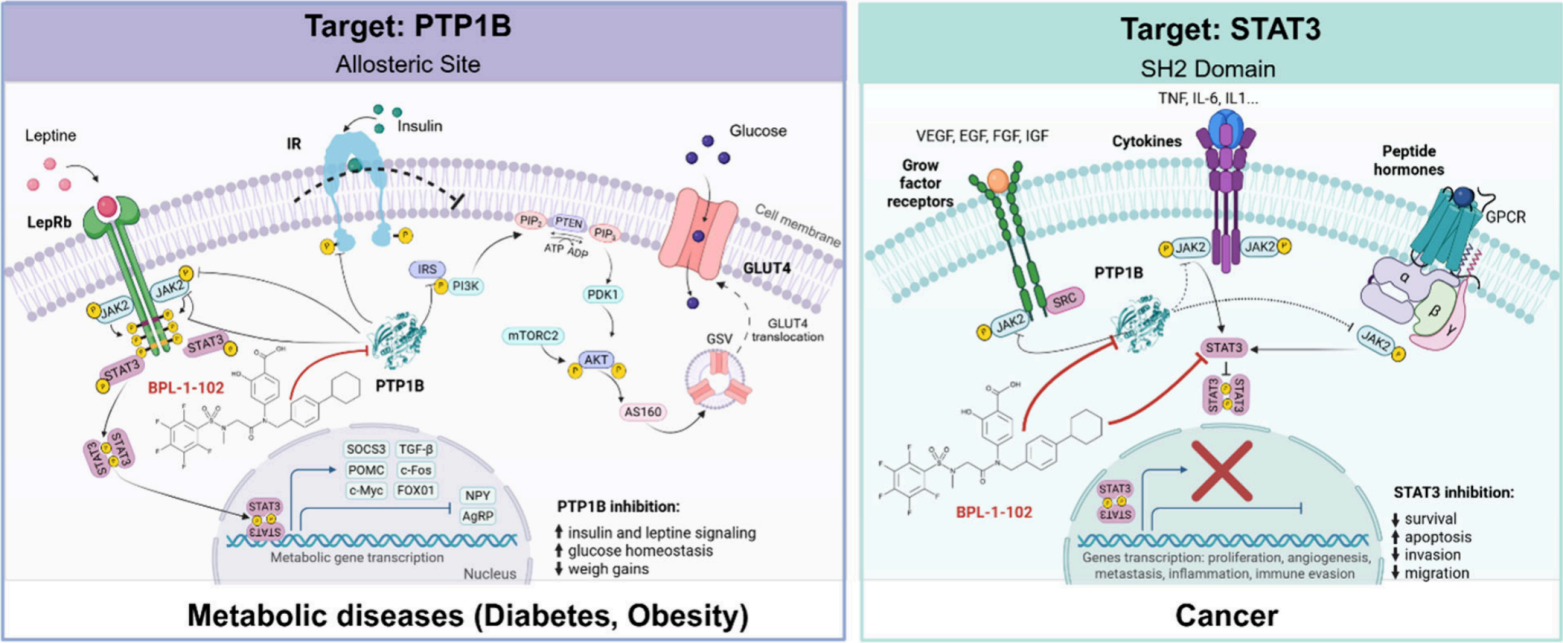
Proposed Dual-Function Mechanism of BP-1-102 at the Intersection of Metabolic and Oncogenic Signaling Pathways.^a^

a (Left) In the metabolic context, BP-1-102 inhibits PTP1B at an allosteric site that bridges the catalytic domain and the intrinsically disordered C-terminal region. This inhibition attenuates the negative regulation of the insulin receptor (IR) and leptin receptor (LepRb) signaling cascades, with downstream effects on glucose homeostasis and transcription of metabolic genes. (Right) In the oncogenic context, BP-1-102 simultaneously targets the STAT3 SH2 domain, blocking STAT3 dimerization and nuclear translocation driven by upstream JAK2, SRC, and growth factor receptor signaling. This action suppresses the transcription of genes governing proliferation, angiogenesis, metastasis, and immune evasion. Collectively, these two mechanisms suggest potential BP-1-102 as a dual-function inhibitor of the PTP1B–JAK2–STAT3 axis, which warrants further evaluation at the interface of metabolic disease and cancer.

By simultaneously targeting the STAT3 SH2 domain and PTP1B, BP-1-102 appears to disrupt this feedback loop at two distinct nodes, effectively bypassing the therapeutic paradox observed with other selective PTP1B inhibitors, such as trodusquemine, where JAK2 restoration inadvertently enhances oncogenic STAT3 signaling. This dual-node interference is particularly relevant in malignancies where both targets are concurrently upregulated, including HER2-positive breast cancer, colorectal and gastric carcinomas, and acute myeloid leukemia, where PTP1B deficiency leads to STAT3 hyperactivation via the CSF-1–STAT3 axis.^33–37^ Supporting this rationale, genetic studies have substantiated that the concurrent loss of PTPN1 and PTPN2 synergistically augments the IL-10/STAT3 axis in T cells, resulting in cooperative antitumor effects that surpass those achieved through single-target inhibition.^31^ Importantly, pharmacological inhibitors of PTPN1 have already demonstrated safety in human subjects and are presently undergoing clinical evaluation as cancer immunotherapy agents (NCT04777994),^32^ thereby further affirming the oncological significance of targeting this phosphatase in scenarios of dysregulated STAT3 activation. In this immunological context, PTP1B has been identified as an intracellular checkpoint in tumor-infiltrating CD8^+^ T cells. Its inhibition has been shown to enhance JAK/STAT5 signaling, thereby promoting T cell expansion and cytotoxicity, as well as potentiating the response to anti-PD-1 therapy.^38^ The dual inhibitory action of BP-1-102 provides a plausible mechanistic explanation for its immunomodulatory effects. This hypothesis necessitates experimental validation in future research.

The favorable passive permeability demonstrated here further supports its cellular accessibility in oncogenic contexts where dual inhibition is most crucial. Collectively, these results propose BP-1-102 as a scaffold for the rational design of dual-target therapy at the metabolic-oncogenic interface and provide the mechanistic and physicochemical basis for its further evaluation in PTP1B/STAT3-overexpressing tumor cell models.

## Safety Statement

No unexpected or unusually high safety hazards were encountered.

## Supplementary Material



## Notes

(Figures S1–S6) Docking protocol validation on PTP1B allosteric site (PDB: 1T49); per-residue Cα RMSF of the C-terminal regulatory region; structural analysis of *h*PTP1B_1–300_-BP-1-102 by MD; individual PCA of *h*PTP1B_1–400_ MD; persistence of ligand–protein contacts; per-residue MM-GBSA binding free energy decomposition; detailed experimental procedures for enzyme inhibition assays; kinetic analysis; molecular docking; molecular dynamics simulations; MM-GBSA calculations; and umbrella sampling (PDF)

## Abbreviations

BP-1-102: 4-(*N*-(4-cyclohexylbenzyl)-2-(2,3,4,5,6-pentafluoro-*N*- methylphenylsulfonamido)­acetamido)-2-hydroxybenzoic acid
*h*PTP1B: human protein tyrosine phosphatase 1B
IC_50_: half-maximal inhibitory concentration
IR: insulin receptor
JAK2: Janus kinase 2
*k*_B_ *T*: thermal energy unit (Boltzmann constant × temperature)
MD: molecular dynamics simulation
MSI-1436: trodusquemine (alternate name)
*R*_g_: radius of gyration
RMSD: root mean square deviation
RMSF: root mean square fluctuation
SH2: Src homology 2 domain
STAT3: signal transducer and activator of transcription 3
TCPTP: T-cell protein tyrosine phosphatase
