# Supplementary material for: Uncompetitive Allosteric Inhibition of PTP1B by BP-1-102 Reveals a Potential Dual-Target Strategy toward the PTP1B–STAT3 Oncogenic Axis: Biochemical and Computational Evidence
Source: ACS Med Chem Lett. 2026 Jun 12;17(7):1440–7. doi: 10.1021/acsmedchemlett.6c00242 (PMC13358984; doi:10.1021/acsmedchemlett.6c00242)
Supplement: Supplementary file 1 [file ml6c00242_si_001.pdf]

Uncompetitive allosteric inhibition of PTP1B by BP-1-102 reveals a potential dual-target strategy toward the PTP1B–STAT3 oncogenic axis: biochemical and computational evidence

Martín González-Andrade[1], Rodolfo A. Lizárraga-Valadez[1], Alejandro Sosa-Peinado[2], Francisco Cortés-Benítez[3] and Nathaly Vasquez-Martínez[1]*

[1]Laboratorio de Biosensores y Modelaje Molecular, Departamento de Bioquímica, Facultad de Medicina, Universidad Nacional Autónoma de México, Ciudad de México, 04510, México.

[2]Laboratorio de Fisicoquímica e Ingeniería de Proteínas, Departamento de Bioquímica, Facultad de Medicina, Universidad Nacional Autónoma de México, Ciudad de México, 04510, México

[3]Laboratorio de Síntesis y Aislamiento de Sustancias Bioactivas, Departamento de Sistemas Biológicos, División de Ciencias Biológicas y de la Salud, Universidad Autónoma Metropolitana - Unidad Xochimilco, Ciudad de México 04960, México.

*Corresponding author. Nathaly Vasquez Martínez, [1]Laboratorio de Biosensores y Modelaje molecular, Departamento de Bioquímica, Facultad de Medicina, Universidad Nacional Autónoma de México, Ciudad de México, CP 04510, México.

E-mail address: nathaly@bq.unam.mx

Table of contents **Page**

A) Supplemental figures S2-S7

B) Experimental procedures S8

C) References S13

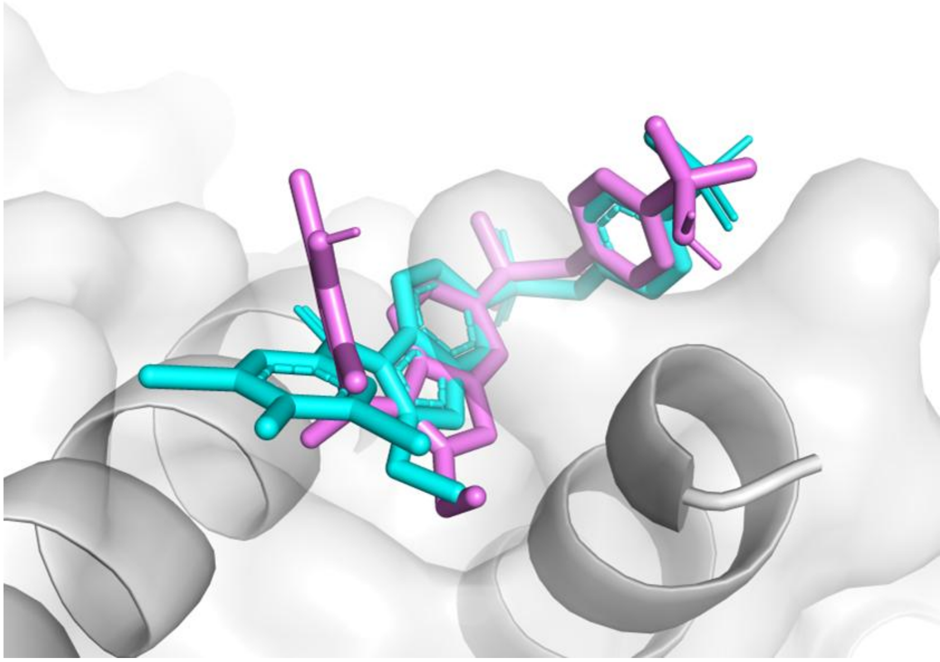

Figure S1. Validation of the docking protocol on the allosteric site of PTP1B (PDB: 1T49). The crystallographic pose is shown in cyan, and the pose reproduced by AutoDock Vina in magenta. RMSD = 1.77 Å.

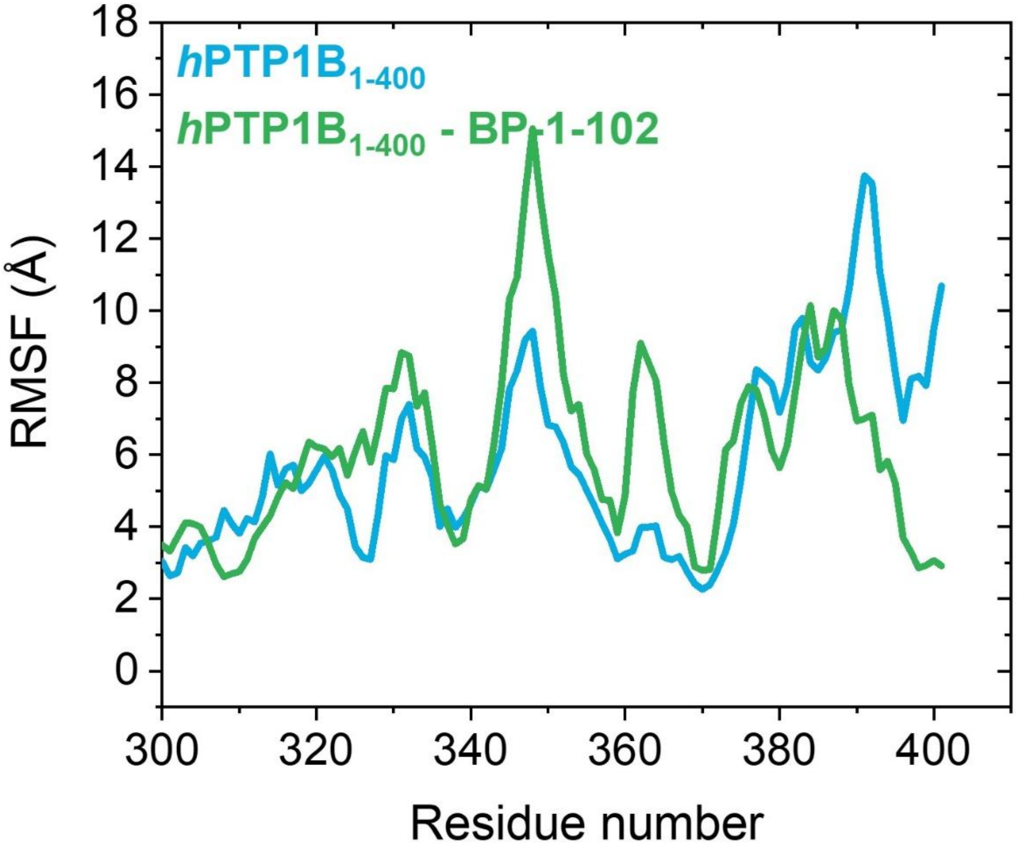

Figure S2. Per-residue Cα RMSF of the C-terminal regulatory region of *h*PTP1B$_{1–400}$. Expanded view of backbone flexibility (residues 300–400) for *h*PTP1B$_{1–400}$ alone (blue) and in complex with BP-1-102 (green) over the 500 ns MD.

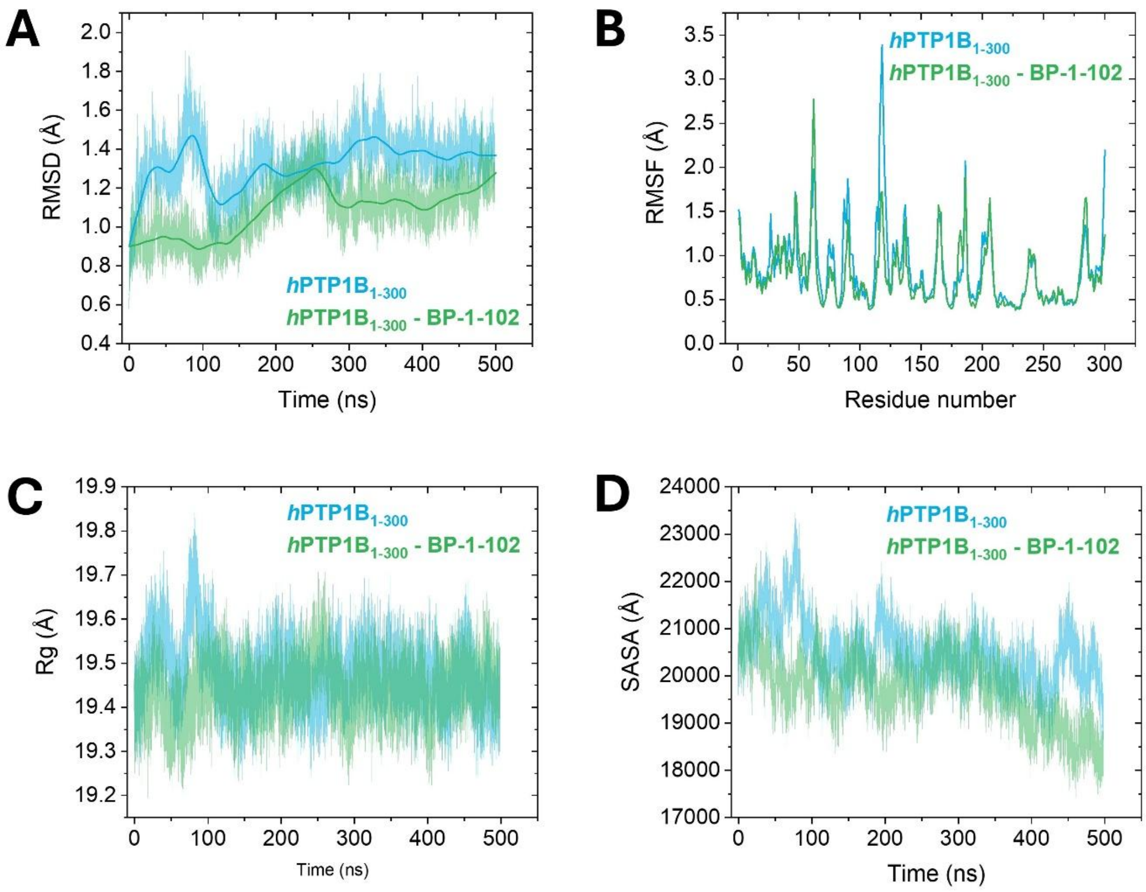

Figure S3. Structural analysis of the *h*PTP1B$_{1–300}$ –BP-1-102 interaction by molecular dynamics simulations**.** A) RMSD, B) per-residue RMSF, C) Rg, and D) solvent-accessible surface area (SASA) of *h*PTP1B$_{1–300}$ alone (blue) and in complex with BP-1-102 (green) over the representative 500-ns MD trajectory.

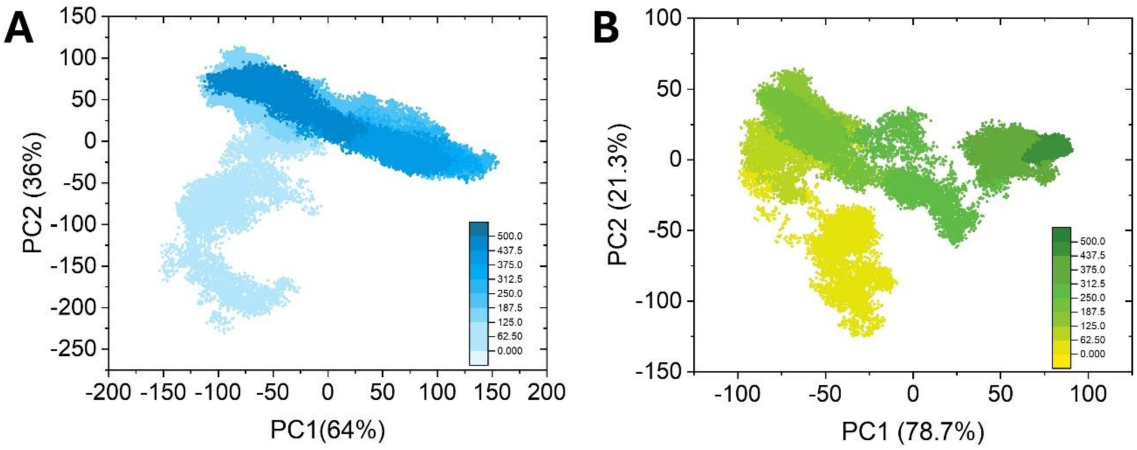

Figure S4. Individual principal component analysis (PCA) of *h*PTP1B$_{1-400}$ molecular dynamic simulations. PCA of backbone atoms (Cα, C, N) projected onto the first two principal components for A) apo hPTP1B$_{1-400}$ and B) *h*PTP1B$_{1-400}$ in complex with BP-1-102 over 500 ns. Color gradients represent simulation time progression from 0 (light) to 500 ns (dark).

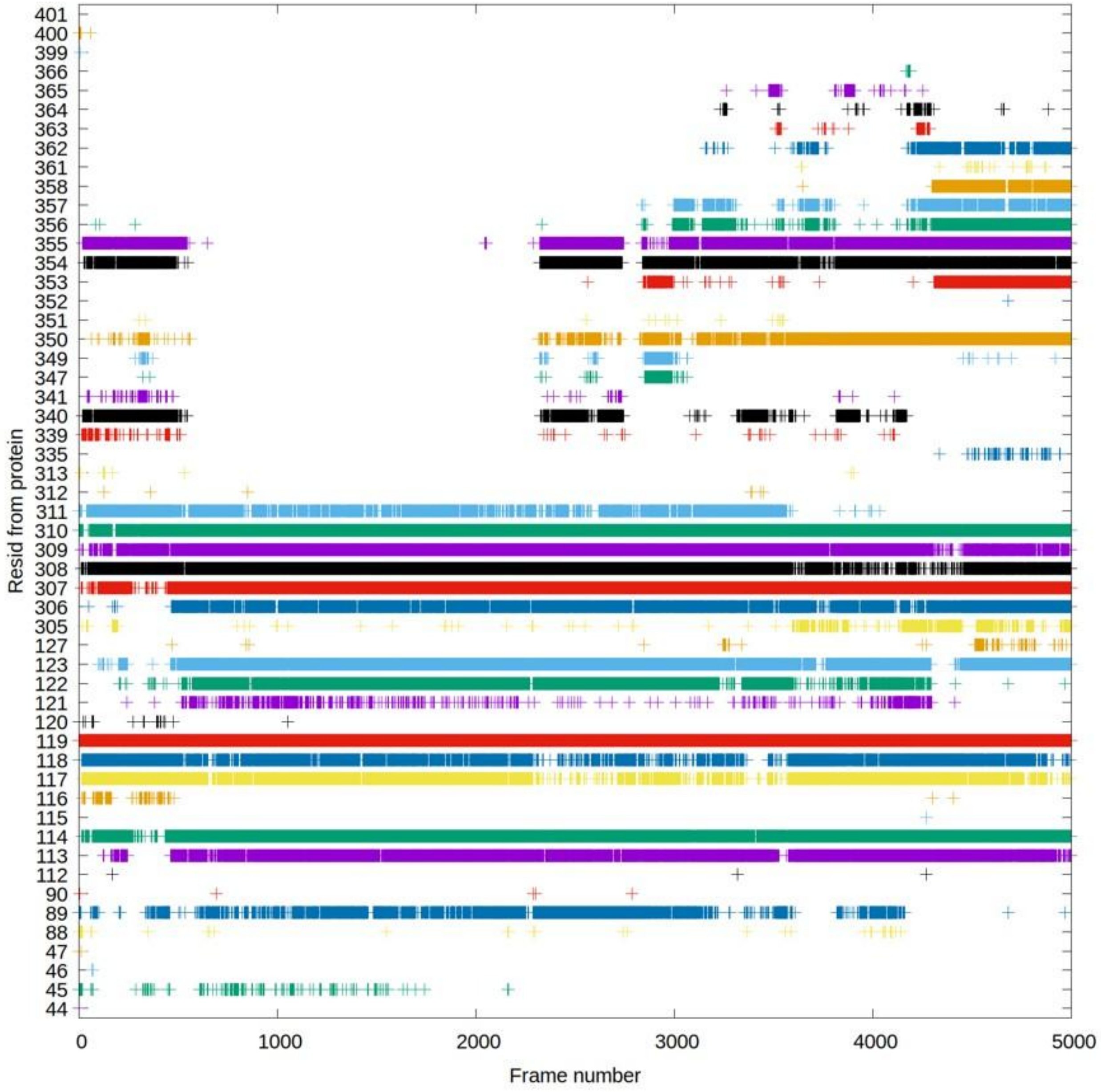

Figure S5. Persistence of ligand–protein contacts throughout the molecular dynamic simulations. Shown are the *h*PTP1B$_{1\text{–}400}$ residues that established at least one contact within a 4 Å cutoff of BP-1-102 during the 500 ns trajectory.

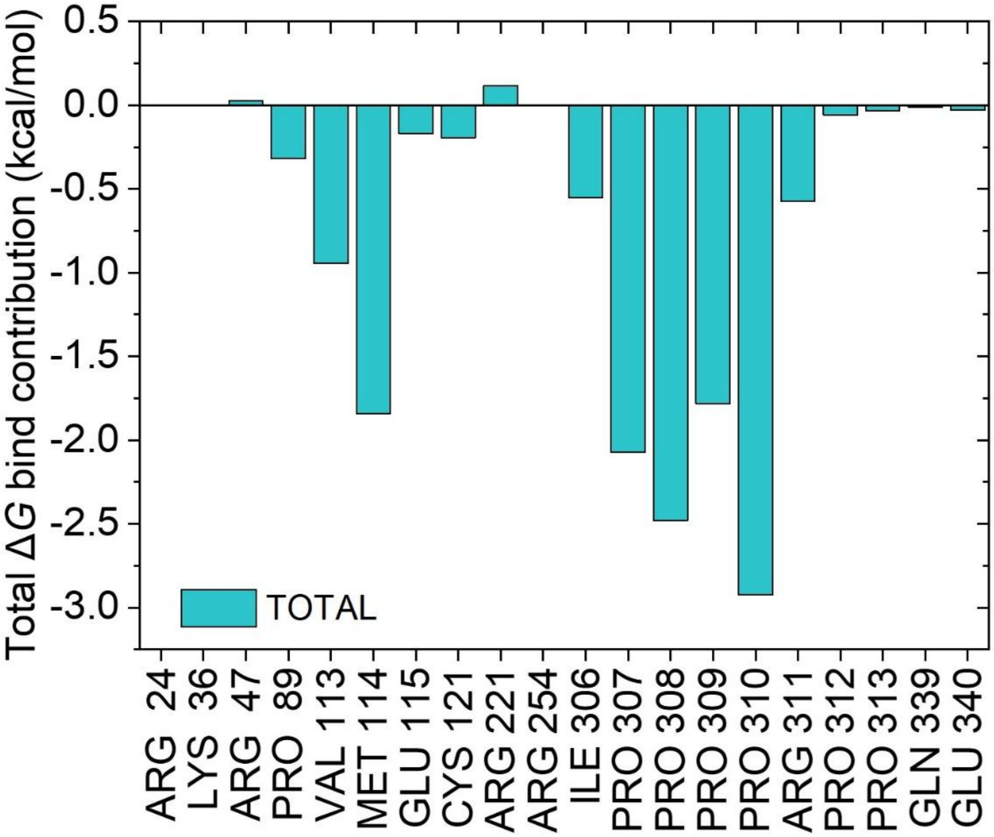

Figure S6. Per-residue binding free energy decomposition of the *h*PTP1B$_{1–400}$–BP-1-102 complex. Total MM-GBSA binding free energy contributions (Δ*G* bind, kcal/mol) for residues showing Δ*G* ≥ 0.1 kcal/mol.

EXPERIMENTAL PROCEDURES

PTP1B and TCPTP inhibition assay

The inhibitory effect of BP-1-102 was evaluated against recombinant human PTP1B (*h*PTP1B$_{1–400}$), *h*PTP1B (residues 1–285). and TCPTP using a previously described spectrocolorimetric assay.[1] Briefly, each enzyme was incubated with 0.5 mM *p*-nitrophenyl phosphate (pNPP) and varying concentrations of BP-1-102 (1–20 µM; TargetMol, USA.) in 50 mM TRIS buffer (pH 6.8). After 15 minutes at 37°C, the absorbance of the hydrolysis product, *p*-nitrophenol (pNP), was recorded at 405 nm using an Accuris SmartReader 96 microplate reader. IC_{50} values were determined via non-linear regression analysis using Equation (1) using OriginPro 2018 (64-bit) SR1 (OriginLab, Northampton, MA, USA):

$$\%\,PTP1B = \frac{A_{100}}{1+\left(\frac{i}{IC_{50}}\right)^{s}} \qquad (1)$$

where *%PTP1B* is the percentage of inhibition, A_{100} is the maximum inhibition, *i* is the inhibitor concentration, IC_{50} is the concentration required to inhibit the enzyme's activity by 50%, and *s* is the cooperative degree. TCPTP activity was assessed under identical assay conditions (50 mM Tris, pH 6.8, 0.5 mM pNPP, 37°C, 15 min, compound concentration range 1–20 µM) as PTP1B, extending to 20–200 µM in absence of inhibition. The determinations were performed in duplicate across at least three independent assays, with results expressed as the mean ± standard deviation.

Kinetic analysis

To characterize the inhibition mechanism, enzyme kinetics assays were performed. Initial reaction velocity was measured at varying substrate concentrations in the presence of increasing inhibitor concentrations, set relative to their IC_{50} value. Data were fitted to the following competitive,

noncompetitive, uncompetitive, and mixed inhibition models mathematically using OriginPro 2018:

Competitive:

$$y = \frac{V_m \times S}{K_m \left(1 + \left(\frac{I_c}{K_i}\right)\right) + S}$$

Noncompetitive:

$$y = \frac{V_m \times S}{\left(1 + \left(\frac{I_c}{K_i}\right)\right) + (K_m + S)}$$

Uncompetitive:

$$y = \frac{V_m \times S}{\left(1 + \left(\frac{I_c}{K_i}\right)\right)\left(\frac{K_m}{1 + \left(\frac{I_c}{K_{ia}}\right)}\right) + S}$$

Mixed:

$$y = \frac{V_m \times S \,/\, \left(\frac{1 + I_c}{\alpha \times K_i}\right)}{\left(S + K_m\left(1 + \left(\frac{I_c}{K_i}\right)\right)\right) \,/\, \left(\frac{1 + I_c}{\alpha \times K_i}\right)}$$

In all models, V_m represents the maximum reaction velocity in the absence of inhibitor, S the substrate concentration, K_m the Michaelis–Menten constant, and I_c the inhibitor concentration. For competitive, noncompetitive, and mixed inhibition models, K_i represents the inhibition constant for inhibitor binding to the free enzyme. For the uncompetitive model, K_{ia} is the inhibition

constant for inhibitor binding to the enzyme–substrate complex. In the mixed model, factor dimensionless alpha (α) describes the relative contribution of each binding mode. Model selection was based on the best fit (adjusted R^2). Additionally, Lineweaver–Burk graphs were constructed to visualize the type of inhibition.

Molecular docking

To study the binding mode of BP-1-102, we used the refined structural model of *h*PTP1B$_{1-400}$ as described and validated in an earlier work.[2] This model incorporates the AlphaFold prediction for PTPN1 (UniProt: P18031; AlphaFold code: Q9PT91), with the C-terminal intrinsically disordered region (residues 300–400) pre-equilibrated via molecular dynamic simulations (MD) to ensure biological relevance for downstream molecular docking and simulations. The 3D structure of BP-1-102 was retrieved from PubChem (CID 53388144). Protein and ligand files were prepared using AutoDockTools 1.5.7,[3] applying Kollman and Gasteiger-Marsili partial charges, respectively. Docking was performed in AutoDock Vina v1.2.5,[4] employing a grid box of $58 \times 126 \times 54$ Å in the x, y, and z dimensions, with central coordinates of 83.4, 72.8, and 32.5 Å. The resulting binding poses were analyzed and visualized using PyMOL (version 2.4.0, Schrödinger, LLC) and Maestro Viewer (v.21.1, Schrödinger, LLC).

The docking protocol was validated by redocking a co-crystallized allosteric inhibitor from PDB entry 1T49 into the prepared receptor. The ligand was extracted from the crystal structure and converted to PDBQT format using OpenBabel v3.1.1 with Gasteiger partial charge assignment. Redocking using AutoDock Vina v1.2.5 within a 20 Å search box centered on the crystallographic ligand centroid (x = 56.02, y = 31.37, z = 22.49 Å). The best-ranked pose was compared to the crystallographic reference by calculating the root mean square deviation (RMSD) of heavy atoms using Open Babel.

Molecular dynamics simulation

To evaluate the dynamic stability of the complexes, ligand topology was generated using Antechamber, while the LEaP module in AmberTools25 was employed to assemble the system.[5]

The protein was parameterized with the ff19SB force field, and the ligand with GAFF.[6, 7] The system was neutralized with Na+ counterions and solvated in a 12 Å TIP3P octahedral box. Minimization was followed by heating to 310.15 K (50 ps), NVT equilibration (50 ps), and NPT equilibration (500 ps). Independent 500 ns MD simulations were performed at 310.15 K and 1 atm, using a Langevin thermostat and a Berendsen barostat for temperature and pressure control, respectively. Periodic boundary conditions and the PME method were applied, while bond constraints were maintained by the SHAKE algorithm (2 fs time step). Trajectories were analyzed using CPPTRAJ,[8] utilizing frames saved every 100 ps for subsequent RMSD, RMSF, and Rg calculations. Principal component analysis and solvent shell analyses were also performed using CPPTRAJ. Binding free energy calculations and per-residue energy decomposition were carried out using the MMGBSA.py module of AmberTools over the 500 ns production trajectory. Graphs were created using Origin 2018. PyMOL and VMD[9] were used to visualize and create MD images.

Umbrella sampling simulations

To evaluate the permeation profile of the compound, the permeation free energy (PFE) profile was calculated using Umbrella Sampling, reconstructed with Weighted Histogram Analysis Method (WHAM) as described previously.[10, 11] A tumor membrane mimetic model with 1-palmitoyl-2-oleoyl phosphatidylcholine (POPC), 1-palmitoyl-2-oleoyl phosphatidylethanolamine (POPE), 1-palmitoyl-2-oleoyl phosphatidylinositol (POPI), and cholesterol was assembled in a 3:3:0.8:3.2 molar ratio[12, 13] using PACKMOL-Memgen and parameterized with the Lipid21 force field.[14-16]. The ligand was initially centered within the bilayer (z ~ 0 Å) and solvated using TIP3P water.

Following minimization, the systems underwent a progressive heating phase from 0 to 100 K (5 ps) and subsequently to 310.15 K (100 ps), followed by a 100 ps NVT equilibration. A steered MD was then performed to define the reaction coordinate by pulling the ligand along the *z*-axis to the aqueous phase (z = 34 Å). From these trajectories, 34 windows were extracted (1 Å spacing), each equilibrated for 1 ns and sampled for 5 ns using 1.25 kcal/mol/Å^2 harmonic restraints. The full symmetric free energy profile (−34 to +34 Å) was obtained by mirror reflection of the unilateral potential of mean force, assuming bilayer symmetry. Simulations were performed in the NPγT ensemble at zero surface tension, utilized a 10.0 Å PME cutoff and were regulated by Langevin and Monte Carlo barostats. All calculations were executed on the GPU-accelerated pmemd.cuda engine in AMBER using an NVIDIA RTX 4090.

REFERENCES

1. De-la-Cruz-Martínez, L.; Martínez-Arellano, R.; López-Sánchez, M.; Alvarado-Rodríguez, J. G.; Torres-Valencia, J. M.; Equihua-González, D., *et al*. Impact of C18 Epimerization of Indole- and Pyrazole-Fused 18β-Glycyrrhetinic Acid Derivatives on PTP1B and TCPTP Inhibitory Activity: Synthesis, In Vitro, and In Silico Studies. *ChemMedChem* **2025;** 20, (22), e202500350. DOI: 10.1002/cmdc.202500350.
2. Díaz-Rojas, M.; González-Andrade, M.; Aguayo-Ortiz, R.; Rodríguez-Sotres, R.; Pérez-Vásquez, A.; Madariaga-Mazón, A., *et al*. Discovery of inhibitors of protein tyrosine phosphatase 1B contained in a natural products library from Mexican medicinal plants and fungi using a combination of enzymatic and in silico methods*. *Front Pharmacol* **2023;** 14, 1281045. DOI: 10.3389/fphar.2023.1281045.
3. Morris, G. M.; Huey, R.; Lindstrom, W.; Sanner, M. F.; Belew, R. K.; Goodsell, D. S., *et al*. AutoDock4 and AutoDockTools4: Automated docking with selective receptor flexibility. *Journal of Computational Chemistry* **2009;** 30, (16), 2785-91. DOI: 10.1002/jcc.21256.
4. Trott, O.; Olson, A. J. AutoDock Vina: improving the speed and accuracy of docking with a new scoring function, efficient optimization, and multithreading. *Journal of Computational Chemistry* **2010;** 31, (2), 455-61. DOI: 10.1002/jcc.21334.
5. Case, D. A.; Aktulga, H. M.; Belfon, K.; Cerutti, D. S.; Cisneros, G. A.; Cruzeiro, V. W. D., *et al*. AmberTools. *Journal of Chemical Information and Modeling* **2023;** 63, (20), 6183-6191. DOI: 10.1021/acs.jcim.3c01153.
6. Wang, J.; Wolf, R. M.; Caldwell, J. W.; Kollman, P. A.; Case, D. A. Development and testing of a general amber force field. *Journal of Computational Chemistry* **2004;** 25, (9), 1157-74. DOI: 10.1002/jcc.20035.
7. Tian, C.; Kasavajhala, K.; Belfon, K. A. A.; Raguette, L.; Huang, H.; Migues, A. N., *et al*. ff19SB: Amino-Acid-Specific Protein Backbone Parameters Trained against Quantum Mechanics Energy Surfaces in Solution. *Journal of Chemical Theory and Computation* **2020;** 16, (1), 528-552. DOI: 10.1021/acs.jctc.9b00591.
8. Roe, D. R.; Cheatham, T. E., III. PTRAJ and CPPTRAJ: Software for Processing and Analysis of Molecular Dynamics Trajectory Data. *Journal of Chemical Theory and Computation* **2013;** 9, (7), 3084-3095. DOI: 10.1021/ct400341p.
9. Humphrey, W.; Dalke, A.; Schulten, K. VMD: visual molecular dynamics. *J Mol Graph* **1996;** 14, (1), 33-8, 27-8. DOI: 10.1016/0263-7855(96)00018-5.
10. Kumar, S.; Rosenberg, J. M.; Bouzida, D.; Swendsen, R. H.; Kollman, P. A. The weighted histogram analysis method for free-energy calculations on biomolecules. I. The method. *J Comput Chem* **1992;** 13, (8), 1011-1021. DOI: https://doi.org/10.1002/jcc.540130812.
11. Vasquez-Martínez, N.; Trapala, J.; Álvarez-Añorve, L. I.; Lizárraga-Valadez, R. A.; González-Andrade, M.; Sosa-Peinado, A. GPU-Accelerated Virtual Screening and Molecular Dynamics Simulations for Identification of Novel DPP-4 Inhibitors. *ACS Omega* **2026;** 11, (4), 5323-5338. DOI: 10.1021/acsomega.5c08231.

12. Szlasa, W.; Zendran, I.; Zalesińska, A.; Tarek, M.; Kulbacka, J. Lipid composition of the cancer cell membrane. *J Bioenerg Biomembr* **2020;** 52, (5), 321-342. DOI: 10.1007/s10863-020-09846-4.
13. Estrada-Perez, A. R.; Bakalara, N.; Garcia-Vazquez, J. B.; Rosales-Hernandez, M. C.; Fernandez-Pomares, C.; Correa-Basurto, J. LC-MS Based Lipidomics Depict Phosphatidylethanolamine as Biomarkers of TNBC MDA-MB-231 over nTNBC MCF-7 Cells. *Int J Mol Sci* **2022;** 23, (20), DOI: 10.3390/ijms232012074.
14. Martínez, L.; Andrade, R.; Birgin, E. G.; Martínez, J. M. PACKMOL: A package for building initial configurations for molecular dynamics simulations. *Journal of Computational Chemistry* **2009;** 30, (13), 2157-2164. DOI: https://doi.org/10.1002/jcc.21224.
15. Schott-Verdugo, S.; Gohlke, H. PACKMOL-Memgen: A Simple-To-Use, Generalized Workflow for Membrane-Protein–Lipid-Bilayer System Building. *Journal of Chemical Information and Modeling* **2019;** 59, (6), 2522-2528. DOI: 10.1021/acs.jcim.9b00269.
16. Dickson, C. J.; Walker, R. C.; Gould, I. R. Lipid21: Complex Lipid Membrane Simulations with AMBER. *Journal of Chemical Theory and Computation* **2022;** 18, (3), 1726-1736. DOI: 10.1021/acs.jctc.1c01217.
